# Stratified medicine in rheumatoid arthritis—the MATURA programme

**DOI:** 10.1093/rheumatology/kew369

**Published:** 2016-12-24

**Authors:** Anne Barton, Costantino Pitzalis

**Affiliations:** 1 Arthritis Research UK Centre for Genetics and Genomics, Centre for Musculoskeletal Research, Manchester Academic Health Sciences Centre, The University of Manchester; 2 NIHR Manchester Musculoskeletal BRU, Central Manchester Foundation Trust, Manchester; 3 Centre for Experimental Medicine and Rheumatology, William Harvey Research Institute, Barts and The London School of Medicine and Dentistry, Queen Mary University of London, John Vane Science Centre, London, UK

Many complex diseases are heterogeneous in their clinical presentation, severity and response to therapies. Understanding this heterogeneity may enable better targeting of treatments to the subgroups of patients most likely to respond—a concept known as stratified (or precision) medicine.

Stratified medicine was identified as a priority by the Medical Research Council (MRC) in 2011, and two consortia from the RA research community applied for funding. In both, the aim was to better predict treatment response in RA. The first reason for focusing on RA was that treatment practice is standardized in England through the National Institute for Health and Care Excellence (NICE) guidelines: MTX is usually the first-choice DMARD; patients who fail to respond to MTX and at least one other DMARD are eligible for biologic drugs, the most commonly prescribed being TNF inhibitor drugs (TNFi); those who fail to respond to first-line biologic therapy can then be switched to the biologic B-cell-depleting therapy rituximab (RTX) [1]. Second, there is a significant non-response rate to MTX (45% by 2 years), TNFi (25% by 6 months) and RTX (40% by 6 months) [2–4]. Third, introduction of early, effective therapy has consistently been shown to improve long-term outcomes, including joint damage, disability and employment [5]. Fourth, studies have shown that response to the first treatment given is the most important predictor of long-term outcome [6]. Finally, while biologics have transformed the treatment landscape of RA, the annual cost to the National Health Service was estimated in 2009 to be ∼£560 M, compared with oral MTX, which costs less than £100/year/patient [7]. The identification of treatment response predictors would allow the allocation of patients to strata defined by the therapy they are most likely to respond to, early in the disease process.

One of the proposals aimed to identify biomarkers in the synovium that could be used as a filter for peripheral blood biomarkers to inform subsequent therapy choices using a prospective biopsy-driven randomized trial approach. The second proposal involved integrated analysis of peripheral blood collected from large cohorts of patients pre-treatment, who are then followed prospectively to assess response in order to identify genetic, genomic and proteomic predictors of response. Both RA proposals were invited to proceed to the full application stage, but the applicants were encouraged to consider whether a combined approach might be more powerful. Following discussion, it was apparent that the overall aims of the studies were similar and the approaches complementary; both recognized the importance of industry involvement and that a combined application would leverage greater benefits than either proposal alone. Hence, the Maximizing Therapeutic Utility in RA (MATURA) consortium was formed. This collaborative approach from the rheumatology research community, involving 12 academic centres and 9 industry partners, was one of the key factors that convinced the funders that the proposal had a high chance of success. Indeed, Arthritis Research UK partnered with the MRC to fund the application after successful competitive interview; MATURA was officially launched in June 2014. Industry partners include large pharmaceutical (Roche/Genentech, Pfizer, AbbVie, MedImmune, Janssen) and diagnostics companies (Beijing Genome Institute, Qiagen), and small medium enterprises (Protagen, Avacta Life Sciences). Their contributions span the funding of study drugs, funding of tests, in-kind generation of data, contribution of data and in-kind bioinformatics support.

MATURA comprises two parallel work-streams (supplementary Fig. S1, available at *Rheumatology* Online). In workstream 1, tissue-driven biomarkers and blood correlates will be investigated in a large synovial tissue biobank from 300 patients (biopsies pre-treatment and 6 months post-DMARD therapy), with associated clinical data collected as part of a previous MRC-funded initiative, Pathobiology of Early Arthritis Cohort (PEAC) [8]. In addition, a prospective biopsy-driven randomized clinical trial will test the hypothesis that discrete cellular and molecular signatures in the synovial tissue (pathotypes) will enrich for response to existing biologic therapies Stratification of Biologic Therapies for Rheumatoid Arthritis by Pathobiology (STRAP).

In workstream 2, large-scale blood-based screening will be undertaken on some of the largest collections and clinical datasets worldwide. Specifically, response to MTX, TNFi and RTX will be investigated in samples collected as part of randomized controlled trials and observational studies in which pre-treatment peripheral blood samples are available for: multi-omic testing, including genome-wide genotyping, DNA methylation and expression profiling using array-based technology; RNA-seq in some samples and a number of proteomic assays. Blood samples are also available at specified time points following treatment initiation in order to allow identification of pharmacodynamic markers of response [9].

Both workstreams are fully integrated through the multi-omic cross-cutting themes that converge in a large analytical and modelling package driven by bioinformatics/statistics experts to develop clinically useful algorithms and companion diagnostics to stratify patients early in disease.

A TranSMART data warehouse platform will be used to store, integrate and analyse the data generated. It has the advantages that it can accommodate phenotypic data as well as high-content multi-omic data; anonymized data can be uploaded by each centre using secure logins, and permissions can be altered to allow different levels of access to different consortium members, dependent on their contribution. A data sharing and security policy document has been developed to ensure that data can be accessed in an equitable, fair and secure manner.

In developing the proposal, the co-applicants worked with local and national patient groups, including INVOLVE, the National Rheumatoid Arthritis Society (NRAS) and Arthritis Care. The Chief Executive of the NRAS attended the final MRC panel interview and provided the patient’s perspective on the value of the tests so as to inform and direct treatment decisions. A patient partner is a member of the Consortium Management board and has been key in developing the strategy for patient and public involvement and engagement. Under her direction, a MATURA Patient Advisory Group has been formed.

The MATURA website [10], developed in collaboration with all partners and patients, contains full details of the studies and samples available; progress to date; the current data analysis plan; and procedures for existing consortium members to propose new analyses and for new consortium partners to join.

In the UK, three other large RA consortia studies have been initiated in recent years (Table 1). In contrast to MATURA (in which the cohorts are defined by the treatment they receive), the other studies have recruited patients with early inflammatory arthritis and aim to identify prognostic and predictive biomarkers of clinical response, remission and resolution. As the patients generally have early disease, it would be anticipated that many will receive MTX, and cross-consortia collaborations are planned.

**Table 1:** Comparison of large consortia studies of RA in the UK

Cohort name	Cohort	Biomedical resource	Access arrangements	Publications arising	Web-site
MAximizing Therapeutic Utility in RA	Prospective longitudinal cohort of established RA with active disease about to start biologic therapy after failure of DMARD treatment; early RA patients about to start treatment with MTX Aim: To identify biomarkers of treatment response to MTX and biologic therapy (anti-TNF, rituximab, tocilizumab)	MTX: *n* = 1500 Anti-TNF: *n* = 1000 Rituximab: *n* = 600 Tocilizumab: *n* = 100 DNA RNA (peripheral blood) Serum Subset with synovial tissue US data	Application documents and process on website	Current manuscript	[9]
Pathobiology of Early Arthritis Cohort	All early arthritis; prospective longitudinal cohort with 3-monthly follow-up data Aim: identify early biomarkers of disease development/progression/resolution	Total in cohort: *n* = 300 Synovial tissue Synovial fluid Serum DNA (peripheral blood) RNA (synovial tissue) US data	Via steering committee	Kelly *et al.* Ann Rheum Dis 2015;74:611–7 [11];Astorri *et al.* Curr Pharm Des 2015;21:2216–24 (review) [12];Hymby *et al.* Arthritis Rheumatol 2015;67:2601–10 [13]	[8]
RA-MAP: Towards a Cure for RA	Prospective longitudinal cohort of early RA Aim: To identify predictors of clinical response and remission in RA patients	Total in cohort: *n* = 275 Serum Plasma Peripheral blood mononuclear cells Whole blood RNA DNA Urine X-rays US data on a subset	Via steering committee	None, to date	[10]
Scottish Early RA Inception Cohort	Newly presenting RA and undifferentiated arthritis; prospective longitudinal cohort with 6-monthly follow-up	On-going collection DNA RNA (peripheral blood) Serum X-ray	Via steering committee	Stalmach *et al.* PloS One 2014;9:e104625 [14]; Kronish *et al.*, Arthritis Rheumatol 2016 (epub ahead of print) [15]	None available

In summary, better targeting of therapies to patients most likely to respond to them, earlier in the disease course, is a fundamental principle underpinning the work of the MATURA Consortium. Although the MATURA work focuses on RA, if successful, similar approaches could be applied to other complex rheumatic diseases for which there is a need for improved treatment response.

## Supplementary data

Supplementary data are available at *Rheumatology* Online.

## Supplementary Material

Supplementary Data
