# Supplementary material for: Stratified medicine in rheumatoid arthritis—the MATURA programme
Source: Rheumatology (Oxford). 2016 Dec 24;56(8):1247–50. doi: 10.1093/rheumatology/kew369 (PMC5850849; doi:10.1093/rheumatology/kew369)

**SUPPLEMENTARY DATA**

**Supplementary Figure S1: Overview of the work-strands and cross-cutting themes forming the work of the MATURA Consortium**


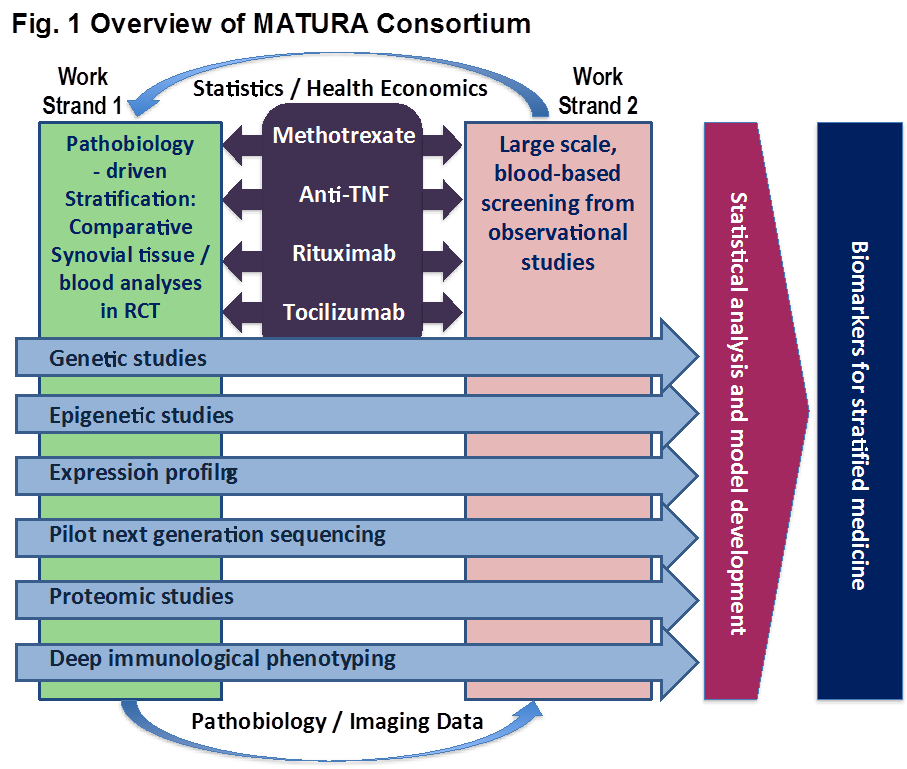

Supplement: Supplementary Data [file kew369_supp.docx]
